# Particle Characterization of Manufactured Sand and Its Influence on Concrete Properties

**DOI:** 10.3390/ma15134593

**Published:** 2022-06-30

**Authors:** Jiale Wu, Weiguo Shen, Deqiang Zhao, Miaomiao Wu, Zhen Yu, Zhicheng Zhao, Zhitang Li, Dinglve Wu, Jiangtao Sun

**Affiliations:** 1 State Key Laboratory of Silicate Materials for Architecture, Wuhan University of Technology, Wuhan 430070, China; jale1355323522@outlook.com (J.W.); deqiangzhao@whut.edu.cn (D.Z.); mmwu@whut.edu.cn (M.W.); 317653@whut.edu.cn (Z.Y.); 317331@whut.edu.cn (Z.Z.); 2 School of Materials Science and Engineering, Wuhan University of Technology, Wuhan 430070, China; 3 Poly Changda Engineering Company Limited, Guangzhou 545000, China; lzhitang@outlook.com (Z.L.); wdinglve@outlook.com (D.W.); sujiangta@outlook.com (J.S.)

**Keywords:** manufactured sand, particle characterization, aggregate imaging measurement system, digital image processing, concrete properties, correlation

## Abstract

With the rapid development of infrastructure construction, it is an inevitable trend to replace natural sand in short supply with manufactured sand to meet sustainable development. In this paper, the relationship between the particle shape characteristics of manufactured sand and concrete performance is discussed using a morphological analysis and concrete experiments. The particle shape parameters of five types of manufactured sand were obtained by using the aggregate image measurement system (AIMS) and digital image processing (DIP) techniques, and the correlations between different parameters were analyzed. Moreover, the properties of concrete with the five kinds of manufactured sand were tested. The results show that particle size and type have a significant impact on particle shape parameters. Particle shape parameters, especially angularity, correlate well with the workability and compressive strength of concrete while having little effect on the durability of concrete. An accurate understanding of the morphological characteristics of manufactured sand is conducive to the optimization of concrete mix designs. Therefore, it is suggested that a manufactured-sand shape test be included in aggregate specification.

## 1. Introduction

Concrete is the most widely used artificial and architectural material due to its unique combination of economic feasibility, availability of critical raw materials, plasticity, mechanical strength, weather resistance, and durability [1,2]. In 2019, the global concrete production was estimated to be 26 billion tonnes [3]. It should be noted that coarse and fine aggregates comprise at least three-quarters of the total volume of concrete [4]. About 32 billion to 50 billion tonnes of aggregates are consumed not only for making concrete but also for making glass, ceramic, mortar, roads, and so on, making sand and gravel the most extracted group of materials, obviously exceeding fossil fuels [5,6]. The unsustainable exploitation and nonrenewable quality of natural sand have almost caused a sand resource exhaustion [7]. Moreover, sand mining on the Pearl River (Zhujiang) in China has lowered water tables, made it harder to extract drinking water, and hastened riverbed scour, damaging bridges and embankments [8,9]. As a result, sand scarcity is an emerging issue with major sociopolitical, economic, and environmental implications, which requires people to find alternative sand resources [6].

Recently, manufactured sand was extensively studied as the main alternative to the natural sand resource [10,11,12]. However, due to its irregular shape, rough surface, highly elongated and flaky particle content, poor gradation, and high stone powder (SP, particle size < 0.075 mm) content, manufactured sand is a low-quality succedaneum of natural sand (NS), and its engineering applications are greatly restricted [13,14,15]. Besides, these features of MS make its applications different from those of NS. Current research mainly focuses on gradation, methylene blue value (MBV), and stone powder content to optimize concrete performance [16]. However, research on the shape of manufactured sand is being left far behind. Although the particle size of manufactured sand is tiny compared to that of coarse aggregate, the number of particles in concrete is enormous. In theory, particle characterization will significantly impact combinations of sands and cement paste, as well as the dispersion of sands in cement paste; thus, manufactured sand morphologies cannot be ignored.

Because of the small size and large quantity of manufactured sand, it is difficult to describe and measure its shape. Up to now, there has been no common standard for the shape description and regulation of manufactured sand [17]. Ueno et al. [18] measured the values of the maximum axis, the minimum axis, and the intermediate axis of manufactured sand with different particle sizes and calculated the ratio of the maximum axis and the middle axis of overall particle size in the way of volume percentage weighting as an index to measure the shape of manufactured sand. Garboczi et al. [19] divided the particle morphology of manufactured sand into four types: disc particles, sphere or cube particles, flake particles, and needle bar particles. However, the definition of each particle shape range in this classification system is too broad to distinguish particles with similar shapes. Cepuritis et al. [13] put the particle parameters of 30 kinds of manufactured sands with different lithologies obtained by a vertical impact crusher into the Zingg diagram. The results showed that the average shape characteristics of all sands obtained by using VSI crushing are highly similar, which means most of the particles are equidimensional, indicating that the influence of the manufactured sand shape of crushing equipment is more significant than the mineral composition of the manufactured sand’s parent rock.

However, the traditional approach of testing aggregate morphological traits is not only time-consuming but also relies heavily on an operator’s subjective assessment, resulting in erroneous and incomplete experimental data [20]. For selecting aggregates and completely understanding the relationship between aggregate morphological qualities and concrete performance, the accurate quantification of aggregate morphological characteristics is essential [21]. DIP technology has gradually become the mainstream method of particle shape evaluation. Shen et al. [22] studied the particle shape and surface roughness of river sand and eight kinds of manufactured sand with DIP technology and found that manufactured sands have higher roundnesses, aspect ratios, and wider parameter distribution ranges compared with river sands. Miller, N.A. [23] used a dynamic image device to study the grain shape of manufactured sand. In the process of particle falling, a digital camera was used to take pictures from 64 directions. The pictures were binarized, and two-dimensional parameters, such as sphericity and aspect ratio, were calculated. Cui et al. [24] measured the morphological characteristics of five aggregates by using the AIMS and investigated the relationship between the single morphological variables of aggregates and the performance of an asphalt mixture. It was found that the angularities or sphericities of the aggregates and the asphalt coverage ratio have strong linear relationships. Wang et al. [25] obtained angularity and roundness through using DIP to investigate the effects of mineral filler morphology on the active adhesion properties between aggregates and mastic. Both roundness and angularity have significant negative relationships with coating ratio, and the correlation between angularity and coating ratio is not as strong as that between roundness and coating ratio.

The latest development in the characterization of manufactured sand particles is the application of 3D measurement technology, such as 3D laser scanning [26], X-ray tomography [27], and scanning electron microscopes [28]. Estephane et al. [29] used the method of combining X-ray tomography and spherical harmonic analysis to characterize the grain shape of six kinds of manufactured sands. After using X-ray tomography, two-dimensional cross-section slices were generated, and then these slices were stacked to obtain three-dimensional images of sand grains. Although the 3D method provides more accurate and abundant particle morphology characterizations, it consumes a significant time and cost. It is impossible to measure small manufactured sand particles due to equipment limitations. As a result, 3D measurement methods are unable to evaluate large volumes of manufactured sand samples quickly and accurately [30]. Although the two-dimensional measurement method is cheaper and easier to operate, it lacks the particle shape parameters that can accurately describe particle shape. Hence, research on the correlation laws between different particle shape parameters and the relationships between particle shape parameters and concrete properties is also insufficient, which cannot effectively explain the influence of manufactured sand particle characterization on concrete performance.

This study evaluated the morphological characteristics of manufactured sand and the relationship between particle shape and concrete performance. The AIMS and improved DIP technology were used to obtain six particle shape parameters, including angularity, to evaluate the morphological characteristics of five kinds of manufactured sands, and the correlations between different parameters were studied. The working performance, mechanical properties, and durability of concrete prepared from these five kinds of manufactured sands with the same mix proportions were tested. Based on the results, the relationships between the shape parameters of manufactured sand and the properties of concrete were evaluated.

## 2. Materials and Methods

**Figure 1:**
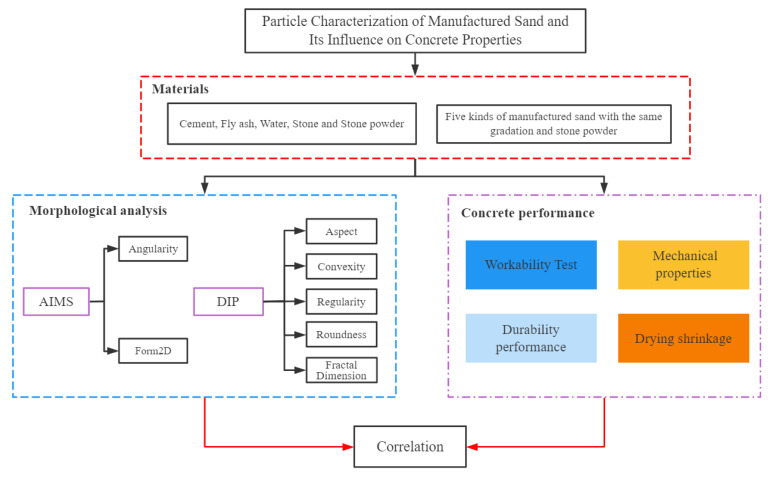
Technical route of this research.

The technical route of this research is shown in Figure 1, and the test details were as follows.

### 2.1. Raw Materials

**Figure 2:**
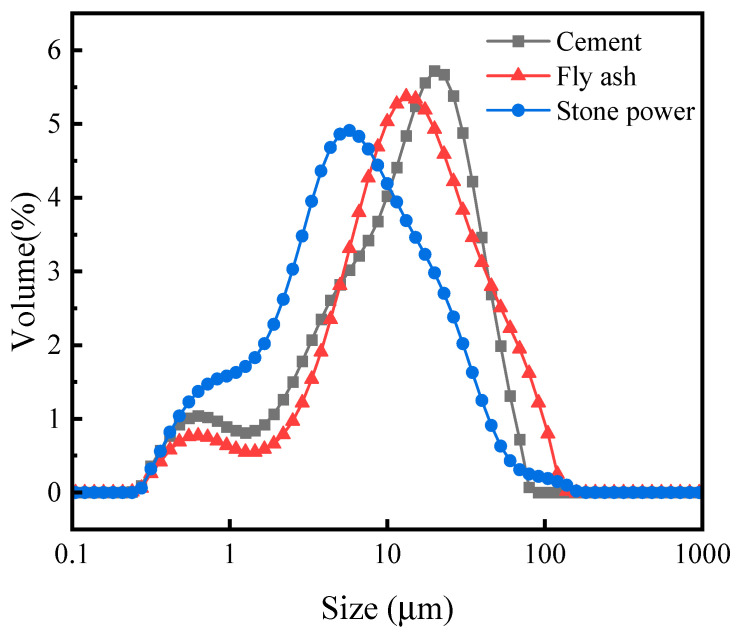
Particle size distributions of cement, fly ash, and stone powder.

**Figure 3:**
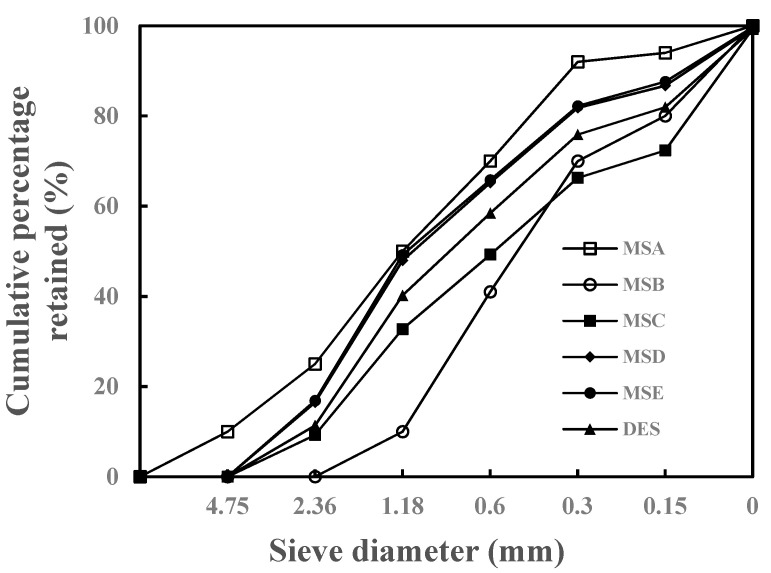
Gradation curves of six sands.

Portland cement was obtained from Cement Company of Guangxi Yufeng. Its characteristics were tested according to GBT1346-2011, and the results are shown in Table 1. Fly ash was obtained from Qingshan Thermal Power Factory, and stone powder was the coproduct of Manufactured Sand A (MSA), which was obtained from dust collectors. The raw materials’ chemical compositions, tested by using X-ray fluorescence spectrometry, are shown in Table 2, and particle size distributions, tested by using laser particle size analyzer, are shown in Figure 2. Coarse aggregate was the re-crushed limestone from digital control cleaner technology, with continuous particle size distribution from 4.75 to 30 mm and an apparent density of 2700 kg/m^3^. Five manufactured sands (MSA, MSB, MSC, MSD, and MSE) were crushed limestone produced from different regions of Guangxi, China, and their physical properties and particle size distributions are presented in Table 3 and Figure 3.

The mixtures of concrete are shown in Table 4. It should be emphasized that the five types of manufactured sand used to prepare concrete were adjusted according to the DES grading shown in Figure 3. All of the stone powers were uniformly replaced by the stone powder in MSA to prevent gradation and MBV from affecting the concrete’s performance.

### 2.2. Method for Measuring Particle Characterization

#### 2.2.1. AIMS

According to GBT14684-2011, 0.5 kg of MS and DMS were taken and sieved, respectively. The test was repeated three times, and the average of different size intervals was taken to make gradation curves.

**Figure 4:**
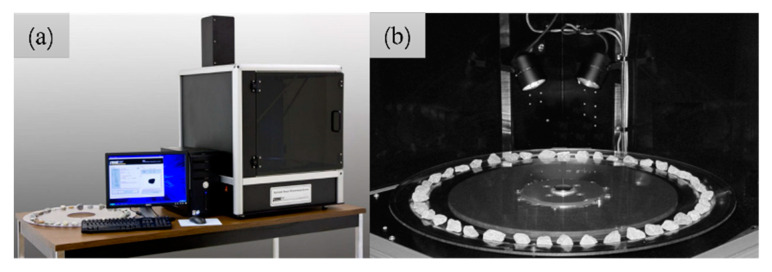
Aggregate image measurement system (**a**) and aggregates being tested (**b**).

The particle shapes of each type of sand were quantificationally characterized using the AIMS (AFA2) produced by Pine Inc., which can acquire accurate fine-aggregate morphology information, such as angularity and form2D, and is used to evaluate the particle morphology characteristics of manufactured sand, the process of which is shown in Figure 4. The angularity is represented by the average change in the gradient vectors, as shown in Equation (1). The angularity value changes in a range from 0 to 10,000; the number is larger as the edge of the aggregate is sharper.

(1) $$\mathrm{Augularity}=1/\left(\frac{n}{3}-1\right)\;\sum_{i=1}^{n-3}|\theta_{i}-\theta_{i+3}|$$

**Figure 5:**
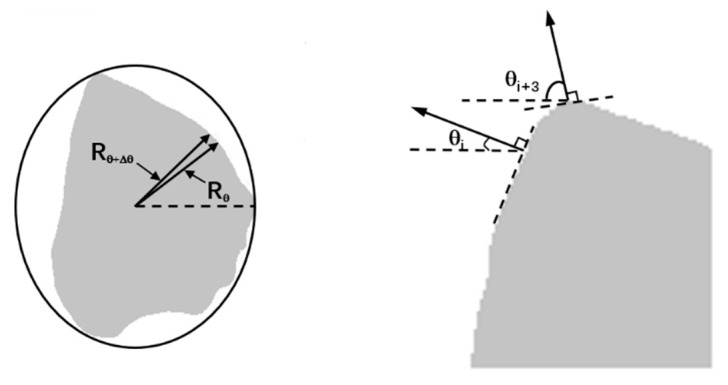
Illustration of form2D and angularity.

*θ* is the angle of orientation of the edge points, *n* is the total number of points, and *i* is the *i*th point on the edge of the particle, as shown in Figure 5.

Form2D quantifies the roundness of fine aggregate on a scale of 0 to 20, and a perfect circle has a 2D value of zero. The calculation of form2D is shown in Equation (2).

(2) $$\mathrm{Form}2D=\sum_{\theta =0}^{\theta =360-\Delta \theta }\left[\frac{R_{\theta +\Delta \theta }-R_{\theta }}{R_{\theta }}\right]$$

*R_θ_* is the radius of the particle at an angle of *θ*, and Δ*θ* is the incremental difference in the angle, as shown in Figure 5.

#### 2.2.2. DIP

This paper adopts the self-designed method of collecting manufactured sand particles to obtain the morphological characteristics of manufactured sand particles accurately and quickly. The main steps are as follows:

(a) After sieving the manufactured sand, clean and dry the particles within the range of 2.36–4.75 mm.
(b) Put the particles on the frame in three different stable placement forms, and take photos from different directions according to needs.
(c) Use Photoshop software to binarize each image.
(d) Analyze each binary image by using Image Pro Plus to obtain five particle shape parameters, such as aspect, convexity, regularity, roundness, and fractal dimension.

Aspect (*As*) represents the ratio of the major axis to the minor axis of the equivalent ellipse of a particle projection diagram. The calculation of aspect is shown in Equation (3):

(3) $$As=\frac{D_{max}}{D_{min}}$$

*D_max_* represents the length of the principal axis of the ellipse and *D_min_* represents the length of the secondary axis of the ellipse.

Convexity (*Co*) is used to reflect the existence and degree of convexity on a particle’s surface, and to a certain extent, it can reflect the size of a specific surface area of a particle. With a convexity approaching 100%, a convex hull area gets closer to the actual area of the particle, and there is less of a concave part in the particle contour. The calculation is shown in Equation (4).

(4) $$Co=\sqrt{\frac{S}{S_{C}}}$$

*S* represents the projected area of the particle, and *S_C_* represents the area of the circumscribed polygon of the particle.

Regularity (Re) reflects the roughness of particle shape, as shown in Equation (5).

(5) $$Re={\left(\frac{P_{C}}{P_{E}}\right)}^{2}$$

*P_C_* refers to the perimeter of the circumscribed polygon, and *P_E_* refers to the perimeter of the equivalent ellipse.

Roundness (*Ro*) indicates the ratio of a circular area to a projected area under the same perimeter as a particle projection. Since the circular area is the largest at the same perimeter, there is always roundness of >1. The closer the roundness value is to 1, the more the particle projection contour is like a circle. The calculation formula is:

(6) $$Ro=\frac{P^{2}}{4\pi S}$$

where *P* is the projected perimeter and *S* is the projected area.

Fractal Dimension (*Fd*) represents the filling degree of a complex shape of a space. The calculation method is:

(7) $$Fd=-\underset{r\to 0}{lim}\frac{ln\;N\left(R\right)}{ln\;R}$$

where *N* (*R*) represents the minimum number of circles required to cover a projection with a circle with radius *R*.

### 2.3. Testing of Concrete Performance

A slump flow test of fresh concrete was performed following GB/T50080-2016. A cone-shaped mold was placed on flat ground and filled with fresh concrete. After removing the mold, the slump was the average diameter of the horizontal spread of the fresh concrete circle.

The compressive strength of DMS concrete was measured by following Chinese standard GB/T50081-2002. The cubic concrete specimens were formed in 150 mm × 150 mm ×150 mm molds. Each mold group was vibrated for 45 s until the concrete became consolidated. After the molds were detached, cubic specimens were cured in a chamber with 100% relative humidity at a temperature of 20 ± 2 °C. At the ages of 7 days and 28 days, concrete samples were tested for compressive strength, and three cubes were tested for each mix proportion and day node.

The durability, including chloride penetration resistance and shrinkage rate of concrete, was measured in accordance with the GB/T50082-2009 standard.

## 3. Testing of Particle Shape Characteristics and Correlation between Shape Parameters

### 3.1. Particle Shape Parameters Obtained by Using AIMS

**Figure 6:**
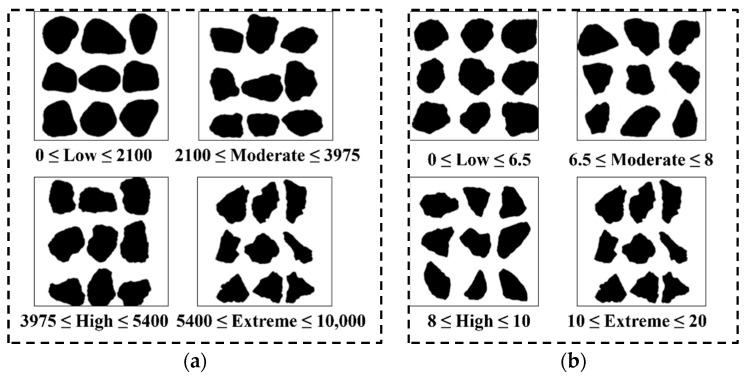
Schematic diagram of angularity and form2D: (**a**) angularity (**b**) and form2D.

An aggregate particle shape can be expressed by three independent indicators, namely angularity, form, and surface texture. The angularity and form2D of manufactured sand can be obtained by using AIMS. The angularity indicates the sharpness of the particle edge, and a stronger angularity brings a larger value. Form2D reflects the regularity of the overall shape of the aggregate. The larger the value, the more slender or flat the particles are, as shown in Figure 6. In this section, AIMS is used to measure the angularity and form2D values of 6 particle sizes of 2.36–4.75 mm, 1.18–2.36 mm, 0.6–1.18 mm, 0.3–0.6 mm, 0.15–0.3 mm, and 0.075–0.15 mm of each kind of manufactured sand to study the influence of the type and size of sand on the shape of manufactured sand.

#### 3.1.1. Angularity

**Figure 7:**
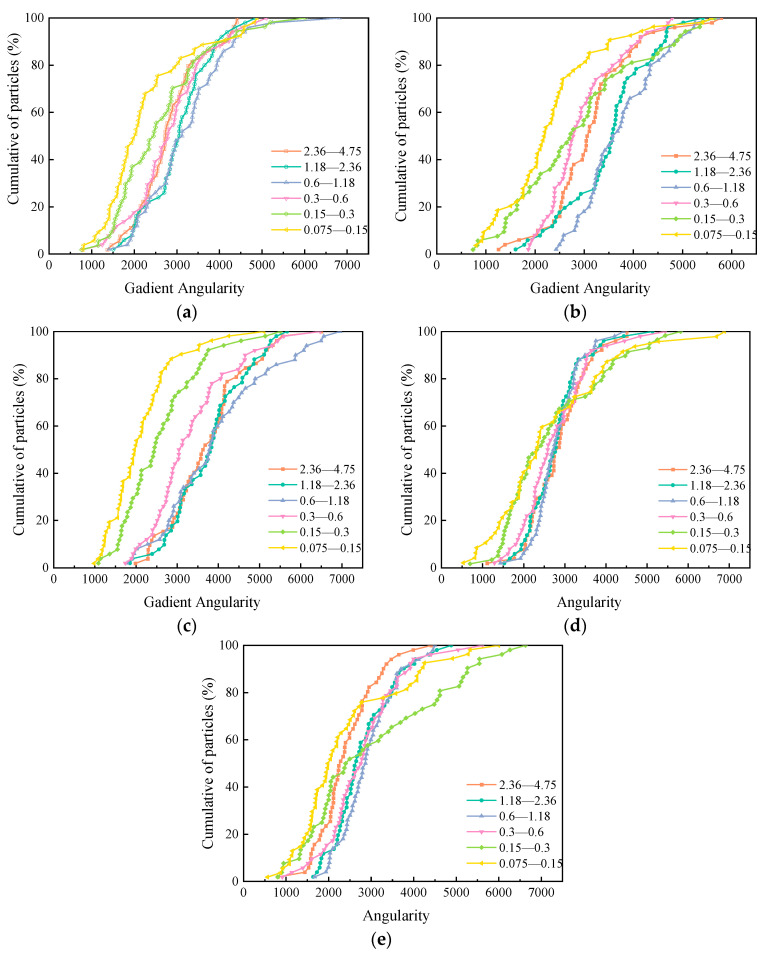
The angularities of five manufactured sands: (**a**) MSA, (**b**) MSB, (**c**) MSC, (**d**) MSD, (**e**) and MSE.

The angularities of the five manufactured sands are shown in Figure 7. The abscissa represents the angular values of the aggregate, and the ordinate represents the cumulative distributions of the aggregate. The closer a curve is to the right, the larger the overall angularity of the aggregate corresponding to the curve. The differences in the angularity curves of the 6 particle sizes of each manufactured sand are obvious, indicating that the particle size of manufactured sand has a significant impact on its angularity.

**Figure 8:**
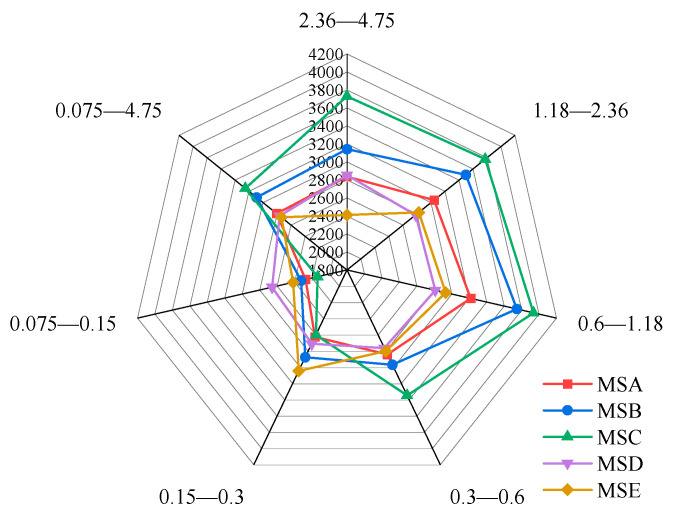
Mean values of angularities of five manufactured sands.

The average values of the prism angle for different grain sizes of sand are shown in the radar diagram in Figure 8. With a decrease in particle size distribution, the angularity of the same kind of manufactured sand shows a trend of first increasing and then decreasing. The maximum angularity is obtained when the particle size range is 0.6–1.18 mm, while the minimum angularity is obtained when the particle size range is 0.075–0.15 mm. The maximum angularity values of MSA—MSE are 41.52%, 61.01%, 84.25%, 5.82%, and 21.18% higher than the minimum angularity values, respectively. This shows that MSC has the largest fluctuation of angularity, while MSD has the most stable fluctuation of angularity among the five kinds of manufactured sand. The angularities of the manufactured sands from different sources are also significantly different. From the perspective of the mean angularity of the whole particle size range, the angularity of MSC is the largest, followed by MSB, MSA, MSD, and MSE. In addition, the angularity within the range of 0.3–0.6 mm is the closest to the average value of the overall particle size, which can be used as a representative of the angularity of each manufactured sand.

#### 3.1.2. Form2D

**Figure 9:**
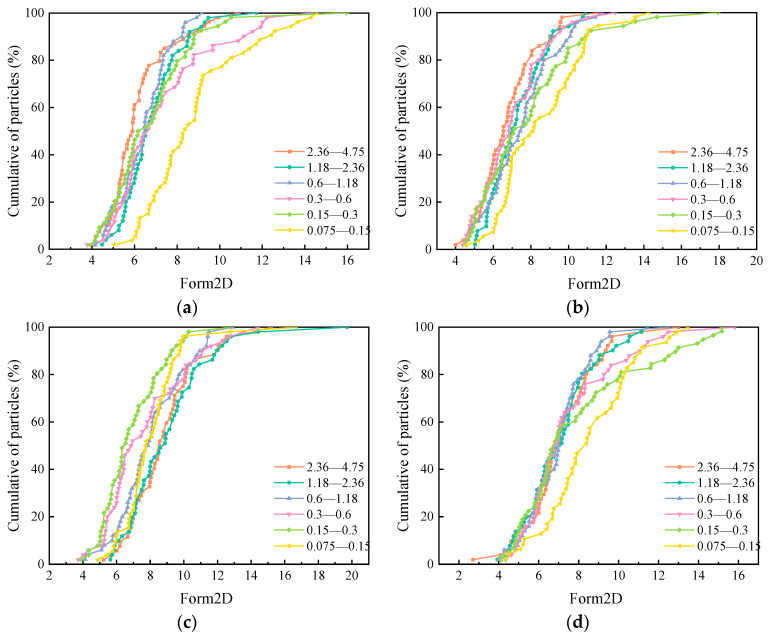

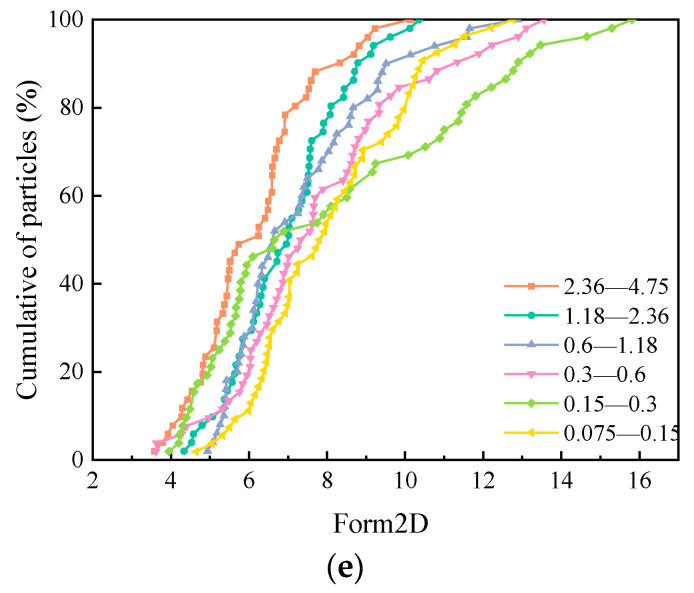
The form2D of five manufactured sands: (**a**) MSA, (**b**) MSB, (**c**) MSC, (**d**) MSD, (**e**) and MSE.

Figure 9 shows the measurement results for the form2D of the five manufactured sands. The abscissa represents the form2D values, and the ordinate represents the cumulative aggregate distributions. The closer a curve is to the right, the larger the form2D of the aggregate, which means that the aggregate is closer to the circle. The form2D of each kind of manufactured sand with different particle sizes is different, which indicates that the particle size of manufactured sand also has a significant impact on the form2D value.

**Figure 10:**
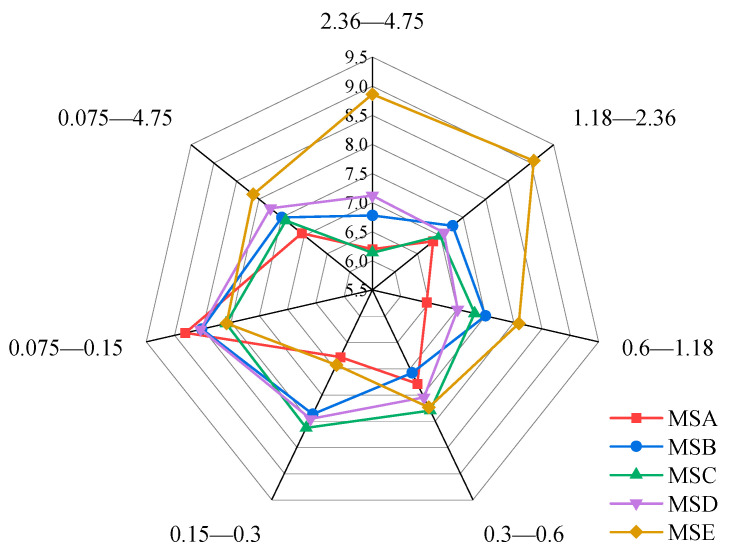
Mean values of form2Ds of five manufactured sands.

The average form2D of each sand is shown in Figure 10. Unlike angularity, there is no clear law on a variation in form2D values concerning particle size. The form2D values of the different manufactured sands in the same particle size range also show different trends. Comparing the average form2D in the range of 0.075–4.75, MSC is the largest, followed by MSD, MSB, MSE, and MSA. Combining angularity and form2D, MSA has the closest two-dimensional shape to a circle and MSC has the most irregular shape.

**Figure 11:**
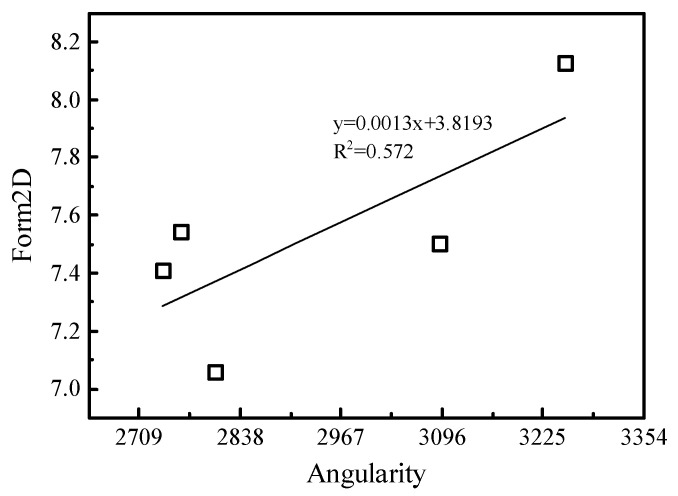
Relationship between angularity and form2D.

Taking the average of the AIMS angularity as the abscissa and the average of the form2D values as an ordinate, the correlation between the two particle shape parameters was studied, and the results are shown in Figure 11. It was found that there is a roughly linear relationship between the angularity and form2D with a poor R^2^ value of 0.572. This proves that angularity and form are two independent indicators. Blott et al.’s [31] research reached a similar conclusion.

### 3.2. Particle Shape Parameters Obtained by Using DIP

**Figure 12:**
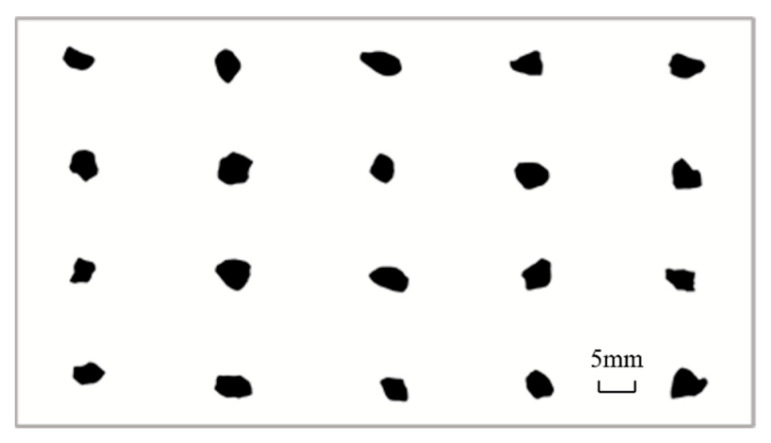
Example of the 20 projections imaged from MSA.

**Figure 13:**
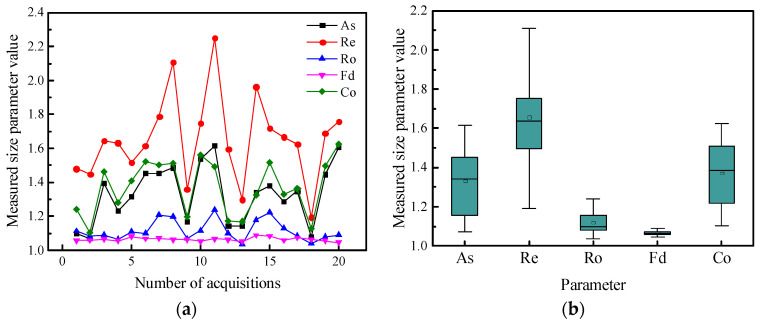
Variations in the parameters measured for the 20 projections of MSA: (**a**) changes in parameters about acquisition direction (**b**) and boxplot showing the variability of the size parameters.

In previous studies, the DIP technology was mainly used to obtain the single-sided projections of aggregates, and multiple acquisitions for 2D image analyses received minimal attention, which was in contrast to an initial objective that academics seek out more information and greater precision values in their research [32]. A grain of MSA was projected 20 times from different directions, and the projection diagram is shown in Figure 12. Then, researchers calculated the size and shape parameters with Image Pro Plus (IPP) software, as shown in Figure 13. It was found that the particle shape parameters obtained by using a single projection have an extensive range of variation and cannot accurately reflect the shape of manufactured sand.

In order to prevent the error caused by single-sided projection, three placement states were adopted for each manufactured sand, three orthogonal projections of each placement state were obtained, and the particle shape parameters of each projection were obtained by using IPP. The aspect, convexity, regularity, roundness, and fractal dimension of each manufactured sand were calculated from the average value of the nine projection images. Fifty particles of each manufactured sand were selected for measurement to accurately reflect the three-dimensional shapes of the manufactured sands through two-dimensional parameters.

**Figure 14:**
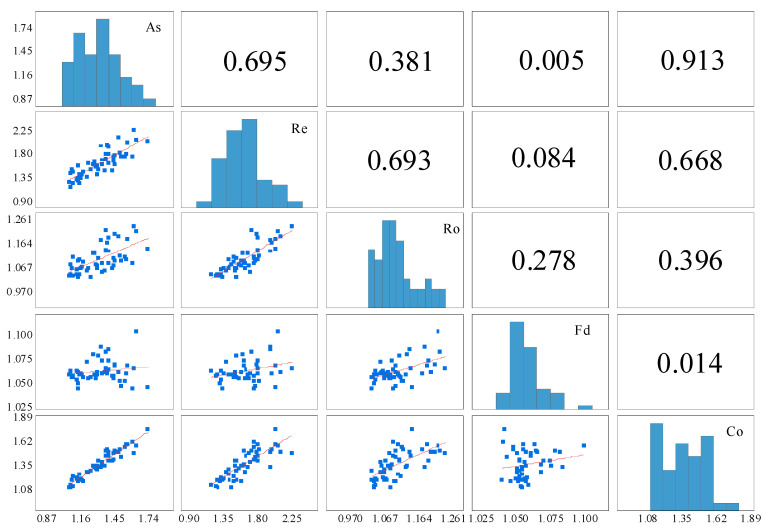
Scatterplots and Pearson correlation coefficients of the particle shape parameters.

Figure 14 is a matrix scatter diagram of several particle shape parameters of MSA. The main diagonal is a distribution histogram of five DIP particle shape parameters. The cell R_ij_ (i represents the number of rows and j represents the number of columns) below the main diagonal represents a scatterplot and fitting line between the two parameters, and the cell R_ji_ above the main diagonal represents the corresponding correlation coefficient. For example, the scatterplot between aspect and roundness can be seen in R_13_, and the correlation coefficient is 0.381, which is shown in R_31_.

As seen from the figures, the particle shape parameters of MSA obey the skew distribution and show an apparent left distribution. There is a specific correlation among the different grain shape parameters, and the correlation between aspect and convexity is the highest with an R^2^ value of 0.913. Convexity has an approximate moderate correlation with other parameters except for fractal dimension, and the correlation coefficient between fractal dimension and other particle shape parameters is minimal.

**Figure 15:**
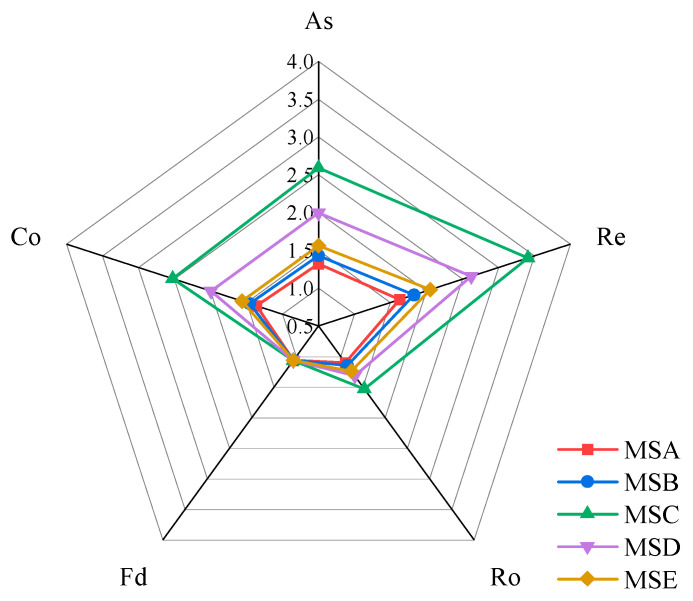
Average values of different DIP shape parameters.

The average values of each parameter were taken in a radar plot, as shown in Figure 15. Except for fractal dimension, the other particle shape parameters all show that MSC is the largest and MSA is the smallest. According to the definitions and physical meanings of these particle shape parameters, when a parameter value is closer to one, a particle has a shape closer to a sphere. It can be considered that MSA has the best particle shape and MSC has the most irregular shape, which is consistent with the results reflected by AIMS. The difference in fractal dimension between the five kinds of manufactured sand is tiny, so it cannot effectively be used to distinguish the particle shapes of manufactured sands. Therefore, the parameter of fractal dimension was not considered in the following correlation analysis.

## 4. Analysis of the Relationship between Particle Characterization of Manufactured Sand and Concrete Performance

### 4.1. Workability of Concrete and Its Relationship with Particle Shape Parameters

**Figure 16:**
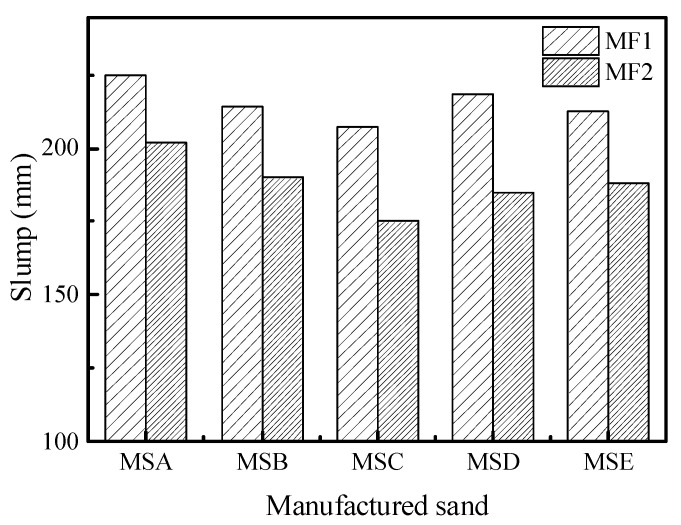
Slump of concrete with different manufactured sands.

Figure 16 shows the workability of the concrete prepared with the five kinds of manufactured sand at two mix proportions. It can be seen that the workability of the concrete was affected by the manufactured sands with different particle shapes. Under the two mix proportions, MSA has the highest slump flow while MSC has the lowest. The slump flow of MSA is 8.05% higher than that of MSC under the MF1 mix proportion and 15.4% higher than that of MSC under the MF2 mix proportion, which indicates that the effect of particle shape is more significant at a low water−cement ratio. This also gives us the idea that when we have to use manufactured sand with poor particles to prepare concrete, we can increase the water−cement ratio to improve the compatibility while ensuring the other properties meet the standards.

**Figure 17:**
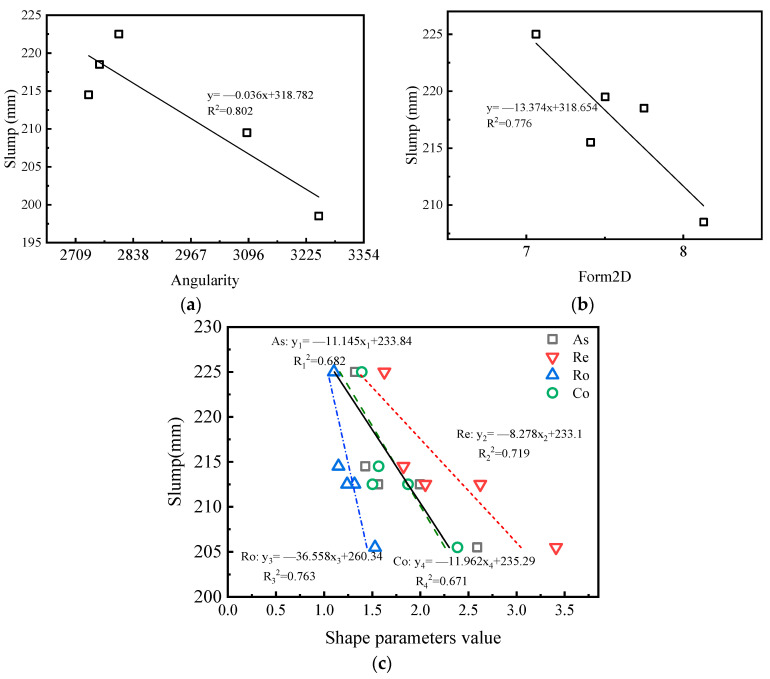
Correlations between particle shape parameters and slumps of concrete: (**a**) angularity, (**b**) form2D, (**c**) and DIP shape parameters.

Figure 17 shows a linear fitting analysis of the particle shape parameters and workability. It can be seen from Figure 17a,b that the workability of the concrete showed a downward trend with an increase in angularity or form2D value. This was because the manufactured sands with multi-edges or irregular shapes increased the specific surface area and consumed more slurry to wrap the aggregate, increasing the interlocking effect and frictional resistance between aggregates and reducing the fluidity of the concrete. It can be seen from Figure 17c that with an increase in several DIP particle shape parameters, the slump of the concrete also showed a downward trend. The correlation between the angularity and the slump of the concrete was the largest with an R^2^ value of 0.802, while the correlation between the convexity and the slump was the smallest with an R^2^ value of 0.671.

### 4.2. Mechanical Properties of Concrete and Concrete’s Relationship with Particle Shape Parameters

**Figure 18:**
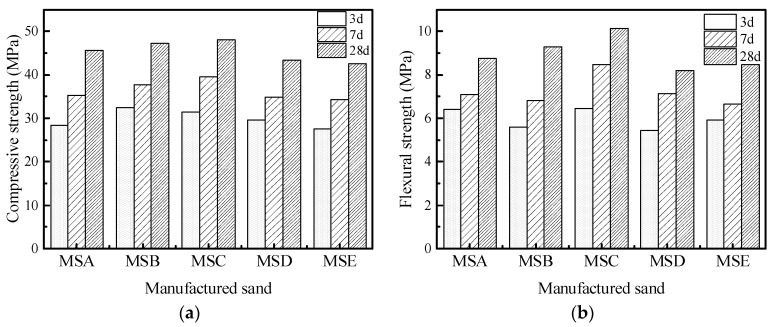
Mechanical properties of concrete under MF1 mix proportion: (**a**) compressive strength (**b**) and flexural strength.

**Figure 19:**
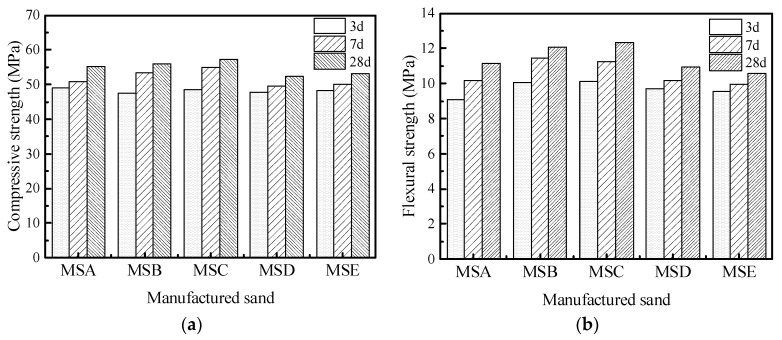
Mechanical properties of concrete under MF2 mix proportion: (**a**) compressive strength and (**b**) flexural strength.

Figure 18 shows the compressive and flexural strengths under the MF1 mix proportion. The compressive strength of MSB is the highest when it is cured for 3 days, while the compressive and flexural strength of MSC is the highest when it is cured for 7 days and 28 days. This shows that the particle shape of manufactured sand has little effect on the early strength of concrete and a noticeable impact on its late strength. Under the MF2 mix proportion, which can be seen in Figure 19, the compressive strength and flexural strength of the concrete show a similar law to that under the MF1 mix proportion. The 28d compressive strength of MSC with the MF1 ratio is 12.95% higher than that of MSE with the lowest strength, and the flexural strength is 23.78% higher, while these two values are divided into 8.03 and 16.9 under the MF2 ratio. This indicates that a decrease in the water to cement ratio or an increase of cementitious material content can reduce the impact of particle defects. A higher amount of cementitious material will also enrich the slurry in concrete, which can fully wrap an aggregate and fill the pores between aggregates to make up for the negative effects of a poorly manufactured sand shape and can improve the strength of the concrete.

**Figure 20:**
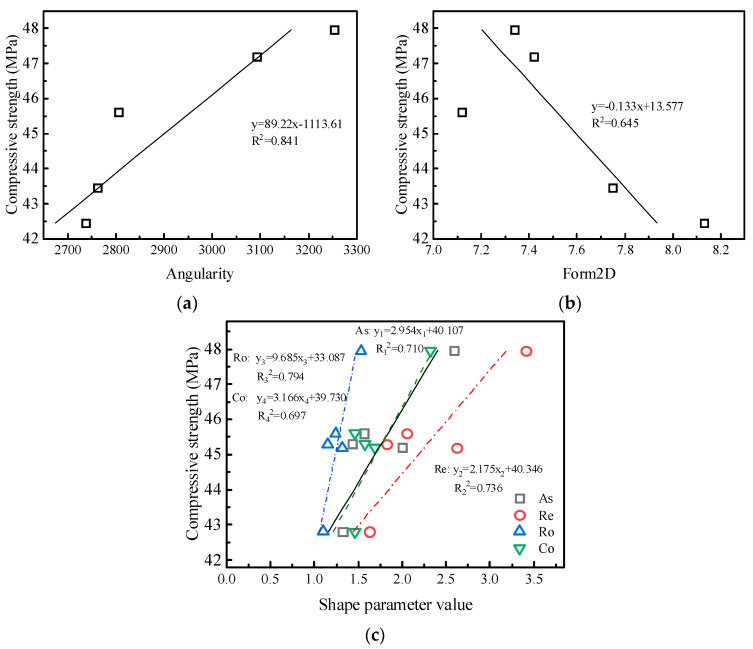
Correlation between particle shape parameters and mechanical properties of concrete: (**a**) angularity, (**b**) form2D, (**c**) and DIP shape parameters.

Figure 20 is a correlation analysis between the 28d compressive strength and particle shape parameters of concrete under the MF1 mix proportion. Each particle shape parameter has different degrees of correlation with the compressive strength. Except for the fact that the form2D value is negatively correlated with the compressive strength, the other parameters are positively correlated. The friction and interlocking effect between the manufactured sand and multi-angular shapes and irregular shapes is stronger to improve the compressive strength of concrete. Among the six particle shape parameters, the correlation between angularity and strength is the largest with an R^2^ of 0.841, and the correlation between convexity and strength is the smallest.

### 4.3. Durability Performance of Concrete and Its Relationship with Particle Shape Parameters

**Figure 21:**
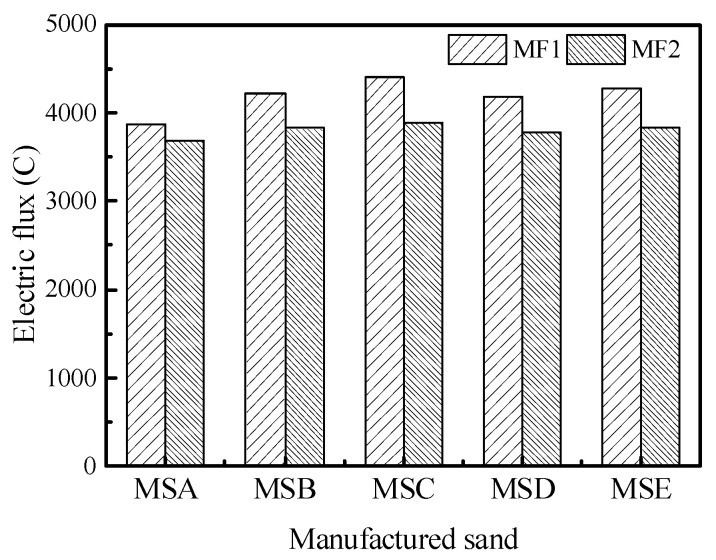
The coulomb electric flux of concrete with different manufactured sands.

**Figure 22:**
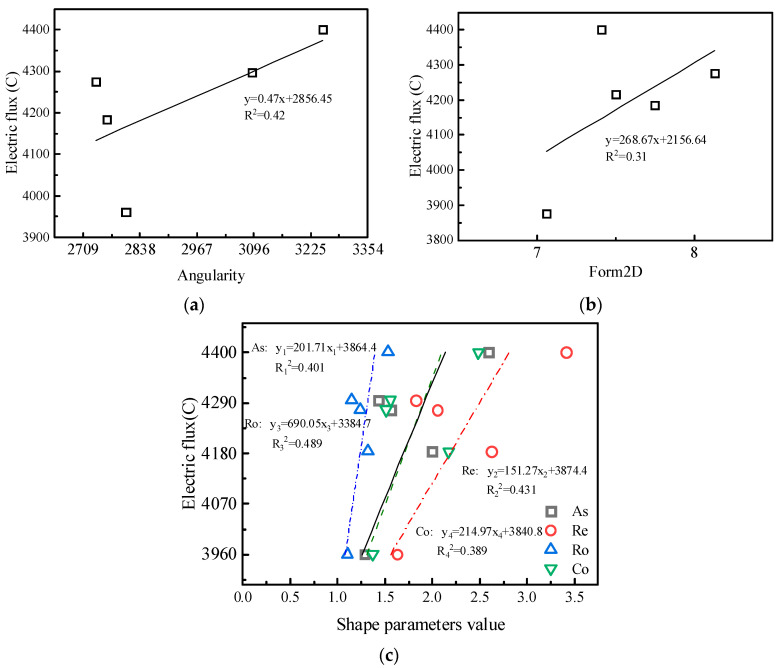
Correlations between particle shape parameters and electric flux of concrete: (**a**) angularity, (**b**) form2D, (**c**) and DIP shape parameters.

The durability of the concrete with the various sands was evaluated using the coulomb electric flux method. Chloride ingress is a major environmental attack in concrete, resulting in the corrosion of rebar and a subsequent decrease in the structural capacity and serviceability of the structural member [33]. The effects of the types of manufactured sand on the electric flux of concrete cured for 28 days under the two mix proportions are shown in Figure 21. Under the two mix proportions, MSC has the largest electric flux and MSA has the smallest, but the gap between the two flux values is not significant. Under the MF1 mix proportion, the electric flux of MSC is only 5.63% higher than that of MSA, indicating that the effect of particle shape on flux is not significant. Figure 22 shows a correlation analysis between electric flux and particle shape parameters. The six particle shape parameters all have an approximately positive correlation with electric flux. With an increase in the DIP particle shape parameter, the particles gradually change to an irregular shape, which also increases the accumulated voids of the particles, thus affecting the impermeability of the concrete. The influence of the DIP particle shape parameters on electric flux is the same as that on compressive strength. However, from the perspective of fitting correlation, the roundness correlation coefficient with the highest correlation among several parameters is only 0.42, and the particle shape parameter cannot sufficiently characterize the change in concrete impermeability.

### 4.4. Influence of Manufactured Sand Shape on Drying Shrinkage of Concrete

**Figure 23:**
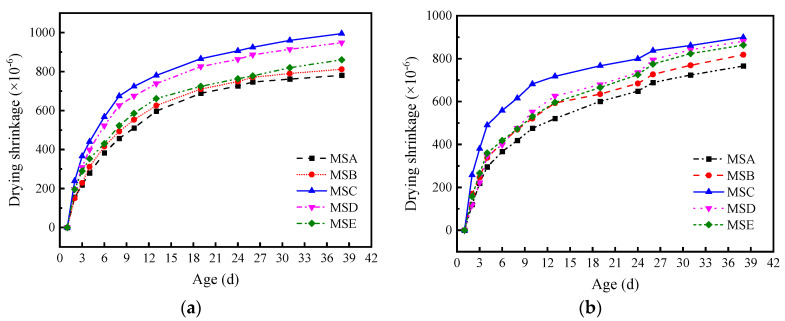
Drying shrinkage strain of concrete with different manufactured sand: (**a**) mix proportion MF1 (**b**) and mix proportion MF2.

Drying shrinkage is one of the main reasons for the cracking of concrete and concrete structures. Figure 23 shows the test results of drying shrinkage. It can be seen that the drying shrinkage rate of manufactured sand increases with an increase in age. The shrinkage of MSC is the highest and the shrinkage of MSA is the lowest under the two mix proportions. MSC, with a high angularity and shape deviating from a circle, has the largest surface area and stacking porosity, which consumes more slurry to wet the aggregate surface and fill the aggregate gap. Under the same drying conditions, it is easier to have large pores after losing water, resulting in concrete shrinkage. The drying shrinkage rate under the MF1 mix proportion is generally higher than that under MF2. For example, the drying shrinkage rate of MSC under the MF1 mix proportion for 38 days is 10.61% higher than that of MF2. This is because more water in the slurry is consumed by the cement hydration process, and the content of free water decreases with an increase in the content of cementitious materials. Moreover, the increase in cementitious materials increases the hydration products of the concrete at various ages, inhibits the volume deformation of the concrete, and leads to a decrease in drying shrinkage.

## 5. Conclusions

Based on different particle characterizations of manufactured sand, the two-dimensional characterization method for manufactured sand shape and its relationship with concrete performance were studied. Combined with the deficiency of DIP single-sided projection, a practical approach for obtaining representative sand information from a two-dimensional image analysis using multiple projections was proposed. The main conclusions are summarized below.

1. The type and particle size of manufactured sand have a significant impact on angularity and form2D. With a range of particle size from large to small, the angularity of manufactured sand increases first and then decreases, while the form2D value has no apparent rules. An angularity within the 0.3–0.6 mm grain size can reflect the mean angularity of the whole grain size of manufactured sand.
2. The particle shape parameters obtained by using image processing with a single projection have an extensive fluctuation range, so it is impossible to accurately judge the particle shape of manufactured sand. More accurate particle shape parameters can be obtained by increasing the number and direction of the projection. There is an obvious linear relationship between the aspect and convexity with an R^2^ value of 0.913, while fractal dimension does not correlate with the other parameters.
3. Under the two mix proportions, concrete with MSA has the best working performance, and MSC has the highest strength and the worst durability. The negative effects of particle shape can be effectively reduced by adjusting the water to cement ratio or the amount of cementitious material as required.
4. The particle shape parameters have good correlations with the slump and compressive strength of concrete, and the correlations of angularity with the slump and compressive strength are the highest, which are 0.802 and 0.841, respectively. The shape of manufactured sand has little effect on the durability of concrete.
5. The particle shape characteristics of manufactured sand have a significant impact on the performance of concrete. An accurate characterization of the grain shape of manufactured sand will be helpful for on-site construction, saving raw materials, and reducing the generation of solid wastes. Therefore, it is necessary to incorporate a particle shape evaluation into the aggregate standard.

## Figures and Tables

**Table 1:** Physical characteristics of cement.

Property	Measured Value
Apparent density (kg/m^3^)	3120
Initial setting time (min)	135
Final setting time (min)	195
Soundness	Qualified
3d Compressive strength (MPa)	27.9
28d Compressive strength (MPa)	52.7
3d Flexural strength (MPa)	6.8
28d Flexural strength (MPa)	9.6

**Table 2:** Chemical compositions of raw materials (%).

Chemical Composition	P·O 42.5	Fly Ash	Stone Powder
CaO	59.55	4.04	39.83
SiO_2_	21.43	3.81	16.47
Al_2_O_3_	5.84	47.12	0.81
Fe_2_O_3_	4.13	33.11	0.52
MgO	3.21	0.61	4.76
SO_3_	2.16	1.44	0.12
Loss	2.39	2.98	37.41

**Table 3:** Physical properties of fine aggregates.

Category	Tap Bulk Density (kg/m^3^)	Void Ratio (%)	Mass Content	Crushed Value (%)	MBV (g/kg)
MSA	1664.3	41.55	12.84	10.85	0.50
MSB	1613.8	42.58	13.46	11.45	0.50
MSC	1516.3	45.69	14.62	14.52	1.50
MSD	1587.6	41.96	15.82	12.58	1.00
MSE	1579.8	43.78	11.46	11.93	0.50

**Table 4:** Mix proportions of concrete (kg/m^3^).

Notation	Cement	Fly Ash	Re-Crushed Stone	Manufactured Sand	Water	Superplasticizer
MF1	312.56	78.14	1086.37	754.93	168.46	3.9072
MF2	411.25	102.81	981.22	740.22	164.52	5.1663

## Data Availability

The data used to support the findings of this study are available from the corresponding author upon request.
